# Wettability of partially suspended graphene

**DOI:** 10.1038/srep24237

**Published:** 2016-04-13

**Authors:** Thierry Ondarçuhu, Vincent Thomas, Marc Nuñez, Erik Dujardin, Atikur Rahman, Charles T. Black, Antonio Checco

**Affiliations:** 1 Nanosciences group, CEMES-CNRS, 29 rue Jeanne Marvig, Toulouse 31055, France; 2 Center for Functional Nanomaterials, Brookhaven National Laboratory, Upton, NY 11973, USA; 3 Condensed Matter Physics and Materials Science Department, Brookhaven National Laboratory, Upton, NY 11973, USA

## Abstract

The dependence of the wettability of graphene on the nature of the underlying substrate remains only partially understood. Here, we systematically investigate the role of liquid-substrate interactions on the wettability of graphene by varying the area fraction of suspended graphene from 0 to 95% by means of nanotextured substrates. We find that completely suspended graphene exhibits the highest water contact angle (85° ± 5°) compared to partially suspended or supported graphene, regardless of the hydrophobicity (hydrophilicity) of the substrate. Further, 80% of the long-range water-substrate interactions are screened by the graphene monolayer, the wettability of which is primarily determined by short-range graphene-liquid interactions. By its well-defined chemical and geometrical properties, supported graphene therefore provides a model system to elucidate the relative contribution of short and long range interactions to the macroscopic contact angle.

Graphene, the one-atom thick, two-dimensional carbon allotrope, has received significant attention owing to its extraordinary electronic, optical and mechanical properties^1^. Advanced coating applications of graphene may also benefit from its high mechanical and thermal stability, excellent chemical resistance and impermeability to gases^2^. Yet, the full technological potential of graphene coatings still requires better understanding of how the atomic monolayer alters the physicochemical properties of the underlying substrate. In particular, the extent of “wetting transparency” of graphene – i.e. transparency to chemical, van der Waals and electrostatic interactions between liquid and substrate atoms or molecules – remains a much debated question^3^,^4^,^5^,^6^,^7^. In principle, the wettability of graphene-coated solids should depend on graphene-liquid short range interactions but also on solid-liquid long range interactions^8^,^9^. An early study by Rafiee *et al.*^3^ suggested that graphene coatings are “transparent” to wetting i.e. do not significantly alter the intrinsic wettability of apolar solids, which interact with water predominantly through van der Waals forces. Conversely, other authors^4^,^5^,^6^,^10^ partially revised these conclusions by showing experimentally that graphene is only partially transparent (or “translucent”) to wetting. Progress on this complex topic has been hampered by experimental shortcomings such as defects occurring during the growth and/or transfer of graphene on a substrate^5^, as well as adventitious carbon contamination^10^, both of which were shown to dramatically alter the intrinsic wettability of graphene and graphitic materials alike^11^,^12^. The theoretical description of graphene wetting phenomena is equally challenging because they are highly dependent on the model taken for the adsorbate-graphene interactions. For instance, the adsorption of water on graphene is not accurately reproduced by density functional theory (DFT) even when effects of dispersive interactions are taken into account^13^,^14^. Several Molecular Dynamics (MD) studies have modeled the wettability of graphene but their results depend quantitatively on the choice of the water−carbon interaction potentials^4^,^5^,^15^,^16^, which are not known precisely. Nevertheless, MD as well as mean field approaches on flat^4^,^5^,^16^ and rough^15^ substrates are consistent with the partial wetting transparency of graphene observed in some experiments.

Despite considerable progress, a comprehensive and consistent understanding of water-graphene interactions is still lacking. Bridging this gap requires the characterization of the intrinsic wetting properties of a suspended graphene sheet in the absence of any interactions with the supporting substrate. However, this is experimentally challenging since capillary forces exerted by macroscopic drops on the graphene membrane may either tear it or fold it. Here, we circumvent this limitation by preparing graphene monolayers partially supported on nanopatterned silicon substrates over macroscopically large (cm^2^) areas. The surface fraction of suspended graphene is varied from 0% to approximately 95% by controlling the morphology of the textured substrate, which allows quantifying the effect of water-substrate interactions on the wettability of graphene. Further, we develop a novel procedure for transferring graphene to a solid support that obviates the irreversible contamination associated to polymer-assisted transfer. The water contact angle on both fully supported and partially suspended graphene depends marginally on the chemical nature of the substrate and the suspension fraction, albeit suspended monolayers are slightly more hydrophobic than supported ones. We show that the wettability of graphene is dictated primarily by water-graphene interactions and to a much lesser extent by water-substrate interactions. By its well-defined chemical and geometrical properties, supported graphene therefore provides a model system to elucidate the relative contribution of short and long range interactions to the macroscopic contact angle^8^,^9^.

## Results

### Fabrication and characterization of suspended graphene layers

**Figure 1:**
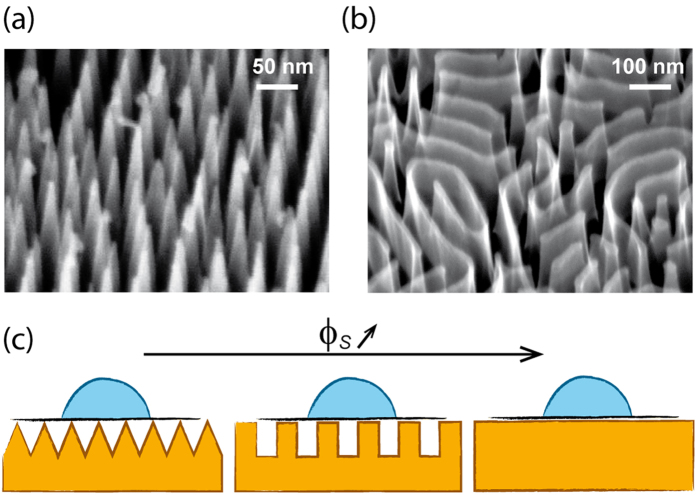
(**a**) SEM micrograph of a nanopatterned substrate with conical tips and spacing of 50 nm. (**b**) SEM micrograph of a substrate with 16 nm-wide grooves and a 70 nm period. (**c**) Sketch of the experiment.

In order to tailor the fraction of suspended graphene, we have fabricated large area (~1 cm^2^), nanopatterned silicon surfaces with uniform feature size and spacing on a 10-nm length scale using block copolymer self-assembly and plasma etching (Fig. 1)^17^. Tapered conical structures with either sharp (width *w* ~ 5 *nm*) or flat (*w* ~ 15 *nm* to *w* ~ 30 *nm*) tips were obtained using a block-copolymer mask with cylindrical morphology and by varying the vertical and lateral etching rates (Fig. 1a). Fingerprint patterns of grooves and ridges (size *w*~12 *nm* to *w*~20 *nm* at the top) were obtained using a block copolymer mask with lamellar morphology (Fig. 1b). A precise control over the texture morphology allowed varying the solid areal fraction at the top of the texture *ϕ*_*S*_ from approximately 5–80%, thereby making these substrates ideally suited to fundamental studies of wetting of suspended graphene by water (Fig. 1c). Moreover, we performed surface functionalization to obtain patterned substrates with either hydrophilic or hydrophobic properties (see Methods for details).

**Figure 2:**
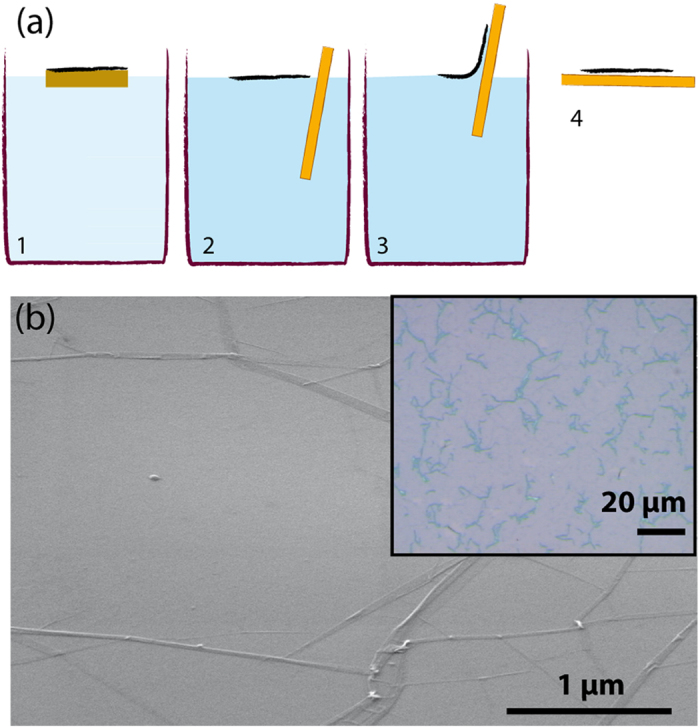
(**a**) Scheme of the transfer method: (1) the copper foil supporting the graphene is etched by an ammonium persulfate solution leaving a floating monolayer (2); the graphene foil is then scooped on a glass slide and redeposited on a water surface for rinsing (not shown); the monolayer is then scooped out on the substrate (3) and dried (4). (**b**) SEM image of a graphene layer deposited on a flat SiO_2_/Si substrate; inset: optical micrograph of the layer; in both images wrinkles appear as darker lines.

Supported and partially suspended graphene monolayers were transferred from copper foils bearing graphene grown by chemical vapor deposition (CVD)^18^,^19^. A most common procedure for transferring graphene from copper to another supporting material starts by stabilizing the supported graphene monolayer with a thick layer of polymer (e.g. poly-methyl methacrylate, PMMA)^3^,^4^,^5^,^10^. Although this method allows transferring large (~1 m^2^) graphene films without compromising its mechanical integrity^20^,^21^, it leads to irreversible polymer contamination of the graphene surface^22^,^23^, thereby altering its intrinsic wettability. In order to circumvent this issue, we have developed a polymer-free transfer method sketched in Fig. 2a and described in detail in Methods section.

The quality of the layers transferred onto flat and nanopatterned silicon substrates was assessed by optical microscopy, atomic force microscopy (AFM), Raman spectroscopy and scanning electron microscopy (SEM). The overall integrity of the graphene layer deposited on flat silicon is preserved during the transfer procedure leaving large areas available for contact angle measurements. However, contrary to polymer-assisted transfer methods, the graphene monolayer exhibits wrinkles most likely caused by surface tension forces acting on the floating layer after the copper foil is etched away (Fig. 2b and Supplementary Fig. SI1). These wrinkles appear as darker, narrow lines (~100 nm-wide) in both SEM and optical microscopy images (Fig. 2b) covering 6-8% of the graphene surface. Moreover, graphene pinholes were observed with diameters of a few micrometers covering about 1-2% of the graphene surface.

**Figure 3:**
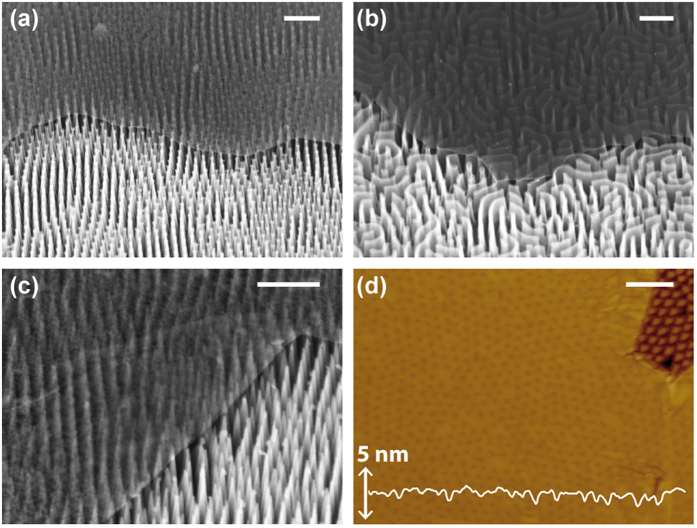
(**a**–**c**) SEM images of graphene layers deposited on textures composed of tapered cones with flat tips, grooves, and tapered cones with sharp tip, respectively; (**d**) AFM image of graphene layer on a conical texture where the white line represent a cross-sectional profile. Scale bar is 200 nm.

Graphene monolayers transferred onto nanopatterned substrates remain suspended without sagging significantly into the voids, regardless of the porosity of the texture, as shown in Fig. 3 (see Supplementary Section SI2 for further details). AFM inspection reveals that the root mean square (rms) roughness of the graphene layer deposited on a nanocone texture is less than 0.4 nm between wrinkles (see inset of Fig. 3d). This remarkable result is understood by considering the large elastic bending energy required to conform the graphene sheet to textures with extremely small period (~50 nm) and high aspect ratio. It is consistent with a recent study showing that graphene remains suspended atop post arrays if the inter-post distance is less than a critical length approximatively equal to 250 nm^24^. At the micrometer scale, the structure of the suspended monolayers exhibits a pattern of folds similar in aspect and area fraction coverage to that observed on graphene transferred on flat substrates. However, a significantly larger pinhole density (~8%) was observed on monolayers deposited on sharp nanocone textures with a substrate fraction *ϕ*_*s*_ < 15% (see Supplementary Fig. SI2). On textured substrates, the occasional presence of small cracks connecting series of posts is also observed. We hypothesize that these cracks are formed by releasing the strain induced by capillary forces during the drying of the textured substrates.

The efficiency of the graphene transfer on hydrophobic textures, is smaller partly because of the turbulences occurring during the addition of isopropanol in water, which sometimes tear the graphene layer into fragments too small for contact angle measurements. The transfer of graphene on conical textures systematically leads to fragmented layers which are only partially suspended and cannot be used for contact angle measurements.

While our graphene monolayers are free of polymer contamination, adventitious carbon readily adsorbs on graphene exposed to ambient air and alters its intrinsic wettability^10^. In order to remove these contaminants, the samples were systematically annealed at high temperature under a continuous flow of reductive Ar/H_2_ atmosphere (see Methods)^22^,^23^,^25^,^26^. We observed that this process effectively removes the adsorbates on the commecial CVD-grown graphene samples. The efficiency of the cleaning protocol was assessed by high resolution transmission electron microscopy imaging and diffraction while Raman spectroscopy gave strong indications of a single monolayer (see Supplementary Section SI3).

The protocol for reproducible contact angle measurement was optimized on graphene monolayers transferred onto flat silicon dioxide from three different commercial CVD-grown graphene sources (see Supplementary Section SI4). Advancing and receding contact angles were measured on several graphene regions, immediately after the reductive annealing and for a few hours afterwards. The quality of the layers transferred onto flat and nanopatterned silicon substrates was assessed^10^,^11^. Interestingly, the advancing contact angle is very reproducible (standard deviation <1°) whereas receding contact angle is more sensitive to defects (standard deviation >9°)^5^. Consequently, we characterized the intrinsic properties of graphene layers by measuring advancing contact angle values obtained within ten minutes after the annealing process.

### Wetting properties of the bare substrates

**Figure 4:**
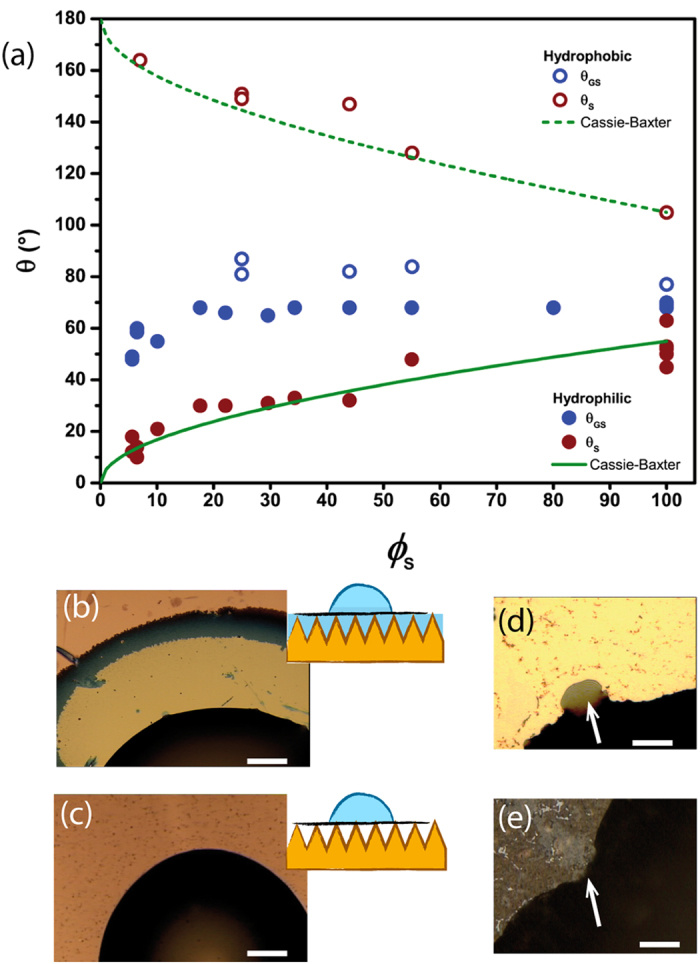
(**a**) Contact angles versus solid area fraction, *ϕ*_S_, for bare hydrophilic (filled red circles), bare hydrophobic (hollow red circles), graphene-coated hydrophilic (filled blue circles), and graphene-coated hydrophobic substrates (hollow blue circles). Green solid and dashed lines represent the Cassie-Baxter contact angle for bare hydrophilic and hydrophobic substrates, respectively. (**b**,**c**) Top-view optical image of a water drop on a graphene coated hydrophilic and hydrophobic nanotexture, respectively, where the insets depict the wetting conditions schematically (scale bar = 100 μm). (**d**,**e**) Close up view of contact line distortions on a graphene coated hydrophilic and hydrophobic nanopatterned substrates, respectively, where arrows mark the defect location (scale bar = 10 μm).

In order to investigate the influence of a graphene layer on the wetting properties of a substrate, we first characterized the wettability of the bare nanopatterned substrates. The measurements were performed on bare parts of the samples supporting graphene to ensure that both situations (with and without graphene monolayer) were subject to identical surface treatments and that wettability differences can therefore be attributed to the graphene influence. In particular, the Ar/H_2_ annealing significantly modifies the wettability of flat SiO_2_ substrates. We have found that the flat silicon samples were completely wet by water after piranha cleaning (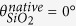) but became less hydrophilic after annealing, 
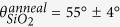
. Conversely, the wettability of the flat fluorinated SiO_2_ samples 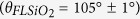 remained unchanged after annealing suggesting that the surface treatment did not significantly compromise the structural integrity of the silane coating. The advancing contact angles of the hydrophilic and hydrophobic nanopatterned surfaces, denoted as 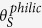
and 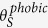
respectively, are reported in red in Fig. 4 as a function of the solid area fraction *ϕ*_*s*_ (red filled and red open circles respectively). These results show that the surface roughness enhances either the hydrophilic or the hydrophobic character of the substrates. For the sharper structures (*ϕ*_*S*_ < 10%), the contact angle was found to be a few degrees for hydrophilic substrates whereas it reached 165° for the hydrophobic ones, typical of super-hydrophilic and super-hydrophobic surfaces^17^, respectively. Optical images of the contact line region revealed that, on hydrophilic nanopatterned surfaces, a wetting film extended from 10 to 100 microns ahead of the contact line, depending on the surface texture. The film appeared bright close to the contact line and dark close to the leading edge. We hypothesize that the film forms through the spontaneous impregnation of the textures with water^27^. The film color variation reflects changes in thickness; the film is thick enough to cover the texture completely in the region close to the contact line, but only partially near leading edge. This “hemiwicking” occurs when the contact angle on the walls is smaller than a critical value defined by 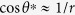 where *r* is the roughness ratio^27^. Our textured samples exhibit spontaneous wicking owing to their relatively high roughness (*r* = 5 − 10), and intrinsic hydrophilicity (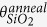 = 55° on annealed SiO_2_). Conversely, no such film was observed ahead of the droplet contact line on hydrophobic textures. In this case, wicking is suppressed by the surface hydrophobicity (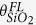 = 105° on fluorinated SiO_2_) and the droplet remains suspended on the texture.

Based on these observations, we have modeled the contact angle of wettability *θ*_*S*_ of the bare patterns, denoted as *θ*_*S*_ using the Cassie-Baxter (CB) equation^28^.


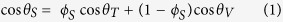


where *θ*_*T*_ is the contact angle on the textured material, and *θ*_*V*_ the contact angle on the medium filling the texture voids, i.e. water (*θ*_*V*_ = 0°) or air (*θ*_*V*_ = 180°) for the hydrophilic and hydrophobic textures, respectively. The CB model is plotted against the data in Fig. 4a using the experimental values *θ*_*T*_ = 55°, 105° for the hydrophilic (solid green line) and hydrophobic (green dashed line) pattern, respectively. The good agreement between theory and experiment suggests that the CB model describes adequately the wetting of the whole range of complex composite surfaces studied here.

### Wettability of partially suspended graphene monolayers

Next, we measured the contact angle *θ*_*GS*_ of water on the graphene layers transferred on the same nanostructures. On hydrophilic substrates, the optical observation of the droplets revealed features similar to the ones obtained on bare substrates resulting from the liquid impregnation of the textured surface beneath the graphene layer ahead of the contact line (Fig. 4b). Evaporation or removal of the droplet with the same syringe used for liquid dispensing demonstrated that the layer below the droplet is also filled with liquid. Under these conditions, the measured contact angle thus reflects the wettability of a graphene layer partially suspended on water, as schematized in the inset of Fig 4b. This contact angle was found to decrease very slightly with decreasing *ϕ*_*s*_ ranging from 
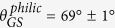
, the value obtained on flat SiO_2_ substrates, to 
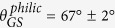
 for textures with *ϕ*_*s*_ < 15%(blue filled circles in Fig. 4a). However, a significantly lower contact angle in the range 
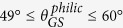
 was found for sharp conical structures (*ϕ*_*s*_ < 15%).

The same experiments were performed on the hydrophobic substrates, where the weak adhesion of graphene onto fluorinated nanopatterned substrates occasionally resulted in graphene lifting off from the surface to wrap the droplet during contact angle measurements^29^,^30^. This issue, combined with the difficulty of transferring graphene to substrates with *ϕ*_*S*_ < 25%, resulted in a smaller number of reliable measurements on hydrophobic samples than on hydrophilic ones. Optical imaging of water droplets deposited on graphene supported by hydrophobic textures showed that the liquid does not spread ahead of the contact line (Fig. 4c) or beneath the graphene layer. This was due to the super-hydrophobic properties of the supporting substrate, which led to a water droplet on a graphene layer partially suspended on air as sketched in Fig. 4c. The contact angle measurements are reported in Fig. 4a in open blue circles. Similarly to the case of graphene supported by hydrophilic textures, no strong dependence on *ϕ*_*S*_ is observed. The average contact angle, was found to increase slightly from 
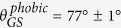
 on a flat fluorinated substrate to 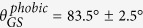 on hydrophobic patterns with *ϕ*_*S*_ = 25%.

We can rule out the possibility that the weak dependence of 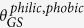 on *ϕ*_*s*_ be due to defects in the graphene layer. In fact, the surface density of defects (either holes and cracks) in supported graphene sheets amounts to ~2% on flat supports and up to 8% on nanocone textures. These defects influence *θ*_*GS*_ depending on the wetting properties of the underlying substrate. On a superhydrophilic substrate, a hole in graphene locally creates a strong wetting defect, whereas on superhydrophobic substrates it gives rise to a strong non-wetting defect. These two types of defects are clearly visible in close-up viewgraphs of the contact line shown in Fig. 4d,e, respectively. Hence, the defects can in principle lead to an apparent decrease of 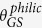 (or increase of 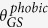) as a function of *ϕ*_*S*_ thereby mimicking the experimental results. We have modelled this effect using the Cassie-Baxter equation (see Supplementary Section SI5 for further details). Our calculations shows that defect densities up to 2% have a limited effect (less than 10%) on *θ*_*GS*_. However, the 8% defect density of graphene supported by superhydrophilic samples (*ϕ*_*S*_ < 15%), may account for up to 30% of the decrease of *θ*_*GS*_ measured on these substrates.

The dependence of *θ*_*GS*_ on the composition of the supporting substrate therefore reflects changes in water-graphene-substrate interactions, rather than the spurious effect of graphene defects. An important finding of this work is that the wettability of graphene varies very little even when it is supported by materials with very different chemical composition such as air, water, silicon dioxide, and fluorinated silicon oxide. This implies that *θ*_*GS*_ is dictated, to a large extent, by water-graphene interactions and, to a lesser extent, by long range water-substrate interactions through the graphene layer.

These findings partially differ from the results of *Raj et al.* who reported no influence of the underlying (hydrophilic) substrate and from a study by Shih *et al.*^4^ who found that graphene is opaque to wetting for hydrophobic substrates (*θ*_*S*_ > 90°) but showed some degree of transparence for hydrophilic substrates (30° < *θ*_*S*_ < 90°). A combined influence of graphene and underlying substrate was also shown^6^,^10^ but not described quantitatively. The lack of consensus among these studies may stem from the choice of contact angle measurement methods (static contact angle is not as well-defined as advancing angle due to contact angle hysteresis) or from sample preparation, which does not systematically eliminate airborne contaminants.

## Discussion

**Figure 5:**
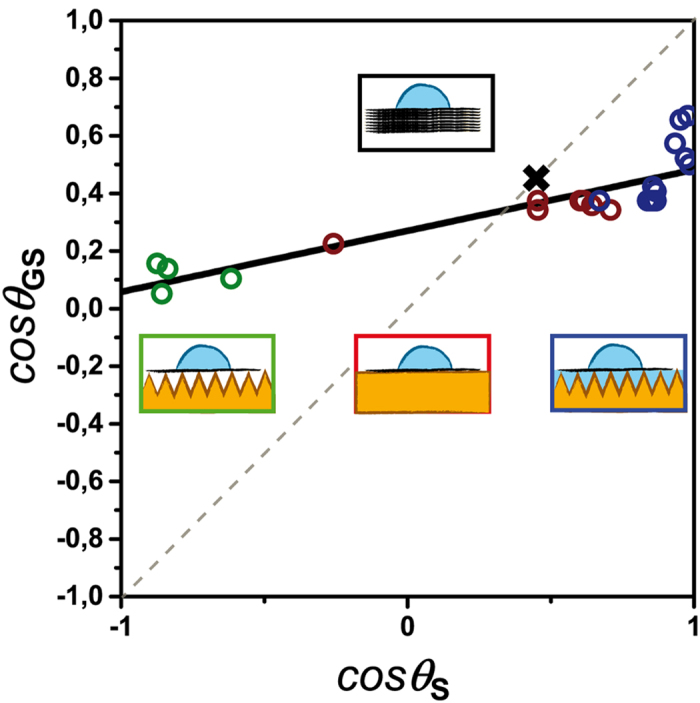
Plot of cos θ_GS_ as a function of cos θ_S_ for the three different systems schematized in the above insets: fully supported graphene in red, partially suspended on air in green and partially suspended on water in blue. The solid black line is a linear fit of the experimental data. Grey dashed line is the cos θ_GS_ = cos θ_S_ line whereas the black cross marks the experimental wetting angle on HOPG.

In order to relate quantitatively the observed wetting translucency of graphene to the underlying molecular interactions, we have plotted the cosine of the contact angle of supported graphene, cos *θ*_*GS*_, as a function of the cosine of the contact angle on the bare substrates, cos *θ*_*S*_. Indeed, cos *θ*_*GS*_ is related to the water-graphene-solid effective interaction potential per unit area *W*_*WGS*_ through the Young-Dupré equation^31^.


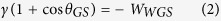


where *γ* is the surface tension of water. Figure 5 gathers the measurements performed on all the fabricated samples which were categorized in three types, namely supported graphene (red open dots), graphene partially suspended on air (green open dots) and water (blue open dots). Remarkably, the data show that all experimental results collapse on a straight line except for data points in a narrow region where cos *θ*_*S*_ ≅ 1. The scattering of data in this region is likely due to the larger density of graphene defects on hydrophilic nanocone textures (*ϕ*_*d*_ ~ 8%).

A linear fit to the data (solid black line) allows extrapolating the water contact angle on two ideal cases: totally suspended graphene *θ*_*G*_ = 85° ± 5° (for cos *θ*_*s*_ =−1), and graphene floating on water, *θ*_*GW*_ = 61° ± 5° (for cos *θ*_*s*_ = +1). Our results are well described by recent mean field calculations of water wetting a flat graphene sheet suspended on a rough substrate assuming dispersive interactions^15^. Specifically, the experimental difference Δ*θ* = *θ*_*GW*_ − *θ*_*G*_ = −24° is in close quantitative agreement with 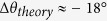. Driskill *et al.*^16^ have predicted a slightly smaller 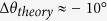 when taking into account both dispersive and dipolar interactions.

The data presented in Fig. 5 are also consistent with the experimental wetting of freshly cleaved, highly oriented pyrolytic graphite (HOPG). Since this material is composed of stacked graphene layers, its wettability should not change with the addition of a graphene coating, leading to the relationship *θ*_*GS*_  =  *θ*_*S*_. Hence, the contact angle of water on HOPG can be determined graphically as the intersection of the linear fit to the data with the cos *θ*_*GS*_ = cos *θ*_*S*_ line (dashed line in Fig. 5). The value *θ*_*HOPG*_ = 70° ± 5° obtained from Fig. 5 is in good quantitative agreement with the experimental *θ*_*HOPG*_ = 62,4° ± 0,9° shown in Fig. 5 as the black cross (see also Experimental Section for details). Wettability of few layered graphene can also be deduced graphically from the data in Fig. 5 as detailed in the Supplementary Section SI6.

The linear relationship between cos *θ*_*GS*_ and cos *θ*_*S*_ can also be understood by writing the generalized Young-Dupré equation for water on the bare substrate: *γ*(1 + cos *θ*_*s*_) = −*W*_*WS*_, water on graphene: *γ*(1 + cos *θ*_*G*_) = −*W*_*WG*_, and water on supported graphene *γ*(1 + cos *θ*_*GS*_) = −*W*_*WGS*_ = −*W*_*WG*_ − α*W*_*WS*_, where *W*_*WS*_ and *W*_*WG*_ are the water-substrate and water-graphene effective interaction potentials per unit area and we have also assumed that *W*_*WGS*_ can be linearly decomposed as *W*_*WGS*_ ≅ *W*_*WG*_ + *αW*_*WS*_. *α* represents a phenomenological “screening parameter” that quantifies the degree of graphene transparency such that *α* = 0describes a perfectly opaque layer. Solving these equations for cos *θ*_*GS*_ yields:


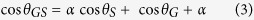


A linear fit to the experimental data gives *α* = 0, 21 ± 0, 03, or *α* = 0, 19 ± 0, 03 when a 2% defect density is taken into account (see Supplementary Section SI5 for further details). This result indicates that graphene screens 81% of the water-substrate interactions compared to a direct contact and is consistent with estimations by mean field theory^32^ leading to about 70% of interactions blocked by a graphene monolayer.

The origin of this screening effect is twofold. On the one hand, the intercalation of graphene between water and substrate increases the average distance between the water molecules and the substrate thereby lowering their interaction. In the case of van der Waals and dipole-dipole interaction the resulting effective interaction potential per unit area then scales as^31^ 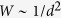. On the other hand, these long range water-substrate interactions are mediated by the graphene sheet. The screening caused by the increased water–substrate distance alone can be approximated as 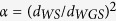 where *d*_*WS*_ and *d*_*WGS*_ are the equilibrium distances between liquid and substrate in contact or separated by graphene, respectively. An estimate of the screening in the particular case of wetting of graphene on water where *d*_*WGS*_ = 2*d*_*WS*_ yields *α* = 0,25. This value is very close to the experimental result, suggesting that, at least for solids and liquids interacting solely through dispersive forces, the “screening effect” can be almost entirely understood as an increase of water-substrate separation upon inserting the graphene coating. Note that, in the general case, the estimation of *α* requires a precise knowledge of the water-graphene and substrate-graphene distances which both are theoretically calculated to be of the order of 3 Å^5^,^33^,^34^.

The experimental value of *α* is smaller than the pure geometrical estimate, which indicates that a small but significant weakening of the water-substrate interactions may arise from the weak but non-zero electrostatic screening efficiency of the graphene layer^35^.

Although these results can be understood qualitatively using continuum models of dispersive and dipolar interactions within a mean field approach, a rigorous quantitative description requires more sophisticated calculations based on DFT and molecular dynamics. We hope that our work will stimulate further theoretical analysis.

## Conclusions

We have presented a comprehensive study of water wettability on graphene suspended on various nanotextured surfaces. By varying the fraction of solid area of the support we were able, for the first time, to measure the water contact angle on a single graphene sheet almost completely suspended on air or supported by water. Through physical and chemical substrates engineering, we were also able to study the substrate dependence of graphene’s wettability to an unprecedented extent. Altogether, these results indicate that the contact angle of water on supported graphene is dictated almost exclusively by (long range attractive and short range repulsive) liquid-graphene interactions. Only ~20% of the long-range interactions between the liquid and the substrate are transmitted through graphene. Our findings shed new lights on the role of liquid-solid microscopic interaction on macroscopic quantities such as the contact angle. They are also relevant to many technological applications of graphene including advanced coatings^36^,^37^,^38^ and water filtration membranes^39^.

## Methods

### Substrate functionalization

The nanopatterned and flat substrates were degreased by sonication in successive baths of acetone, isopropyl alcohol and water. The samples were then immersed in a 40 mL mixture of hydrogen peroxide and sulfuric acid (1:3 v/v) for 15 minutes, thoroughly rinsed with deionized water and dried with nitrogen. This surface treatment results in highly hydrophilic substrates.

In order to obtain the (super)hydrophobic substrates, the substrates were left overnight in a mixture of 10 mL hexadecane, 1 mL choloroform and 133 μL of 1H-1H-2H-2H-perfluorodecyltrichlorosilane (ABCR, Germany) under Ar atmosphere. The substrates were then rinsed in chloroform and dried with nitrogen.

### Graphene transfer method

In our investigations we have used commercially-available graphene monolayers (Graphene Supermarket Inc. USA and Graphenea, SP) grown on copper surfaces by chemical vapor deposition (CVD). A ~1 cm^2^ as-synthesized piece of graphene-coated copper foil is floated at the surface of a dilute aqueous solution of copper etchant ((NH_4_)_2_S_2_O_8_) with graphene exposed to air (see Fig. 2a). A large solution volume (100 mL) and low etchant concentration (10^−2^ M) were used to promote a slow, steady etch rate (<500 nm/h) necessary to prevent the fragmentation of the copper foil into sub-millimeter grains, which may tear and sink the floating graphene layer. After complete dissolution of the copper foil (48–72 h), the graphene monolayer is left floating intact at the liquid-air interface. Although this process is performed in cleanroom environment, adventitious contamination on CVD-grown graphene typically provides enough reflective contrast to see the monolayer floating on the etching solution with the naked eye. The floating graphene is then carefully scooped out onto a rinsing bath of deionized water using a glass slide pre-cleaned in a mixture of hydrogen peroxide and sulfuric acid (1:3 by volume). After typically 15 minutes, the graphene layer is again scooped out of the rinsing bath using the final substrate. In the case of flat and patterned hydrophilic substrates, this step is greatly facilitated by the solution that wets the substrate completely. However, the deposition on hydrophobic substrates is more challenging because water spontaneously dewets these surfaces. We obviated this issue by adding a small amount of isopropanol (12% v/v) to the rinsing bath of distilled water thereby lowering the surface tension of the solution enough to induce complete wetting on the hydrophobic surfaces. After the transfer was completed, the samples were dried at room temperature.

### Graphene cleaning procedure

Prior to any contact angle measurements, the samples were cleaned by annealing under a Ar/H_2_ atmosphere. The samples were heated up to 350 °C following a ramp of 5 °C/min, under a argon flux of 300 sccm, and kept at this temperature during 4 hours with an additional flux of hydrogen (75 sccm). The oven was naturally cooled down to ambient temperature under Ar flux.

### Sample characterization

The samples were first characterized using an optical microscope (Olympus BX60) and scanning electron microscope (FIB-SEM Zeiss 1540XB). Micro-Raman spectra were acquired on a Horiba Xplora-MV2000 spectrometer. AFM characterization were performed on a Multimode 8 AFM (Bruker) in Tapping mode using OTESPA cantilevers. The contact angle measurements were performed on a Kruss DSA100 goniometer following the procedure detailed in Supplementary Section SI4.

### Wettability of HOPG

The wettability of HOPG was characterized using a 10 × 10 × 1 mm HOPG (type ZYA) sample purchased from Scientec (France). The sample was exfoliated several times using a scotch tape until a flat surface was obtained. All measurements were performed on freshly exfoliated surfaces *i.e.* within 5 minutes after the last peeling. The obtained values were reproducible leading to *θ*_*HOPG,adv*_ = 62, 4° ± 0, 9° and *θ*_*HOPG,rec*_ = 60, 2° ± 1, 1°.

When left overnight under ambient conditions, the contact angles drastically changed to reach *θ*_*HOPG,adv*_ = 90° ± 1°, 6° and *θ*_*HOPG,rec*_ = 51, 2° ± 1, 5°. This evolution gives a large increase of hysteresis that can be associated to the adsorption of airborne contaminants, similar to the ones affecting measurements on graphene monolayers.

## Additional Information

**How to cite this article**: Ondarçuhu, T. *et al.* Wettability of partially suspended graphene. *Sci. Rep.* **6**, 24237; doi: 10.1038/srep24237 (2016).

## Supplementary Material

Supplementary Information

## References

[b1] Soldano C., Mahmood A. & Dujardin E. Production, properties and potential of graphene. Carbon 48, 2127–2150 (2010).

[b2] Nine M. J., Cole M. A., Tran D. N. H. & Losic D. Graphene: a multipurpose material for protective coatings. Journal of Materials Chemistry A 3, 12580–12602 (2015).

[b3] Rafiee J. *et al.* Wetting transparency of graphene. Nature Mat. 11, 217–222 (2012). 10.1038/nmat3228 22266468

[b4] Shih C.-J. *et al.* Breakdown in the Wetting Transparency of Graphene. Phys. Rev. Lett. 109, 176101 (2012). 23215205 10.1103/PhysRevLett.109.176101

[b5] Raj R., Maroo S. C. & Wang E. N. Wettability of Graphene. Nano Lett. 13, 1509–1515 (2013). 23458704 10.1021/nl304647t

[b6] Peng S., Lohse D. & Zhang X. Microwetting of Supported Graphene on Hydrophobic Surfaces Revealed by Polymerized Interfacial Femtodroplets. Langmuir 30, 10043–10049 (2014). 25087703 10.1021/la5022774

[b7] Parobek D. & Liu H. Wettability of graphene. 2D Mater. 2, 032001 (2015).

[b8] Bonn D., Eggers J., Indekeu J., Meunier J. & Rolley E. Wetting and spreading. Rev. Mod. Phys. 81, 739–805 (2009).

[b9] de Gennes P. G. Wetting - Statics and Dynamics. Rev. Mod. Phys. 57, 827–863 (1985).

[b10] Li Z. *et al.* Effect of airborne contaminants on the wettability of supported graphene and graphite. Nature Mat. 12, 925–931 (2013). 10.1038/nmat3709 23872731

[b11] Kozbial A. *et al.* Understanding the intrinsic water wettability of graphite. Carbon 74, 218–225 (2014).

[b12] Martinez-Martin D. *et al.* Atmospheric contaminants on graphitic surfaces. Carbon 61, 33–39 (2013).

[b13] Voloshina E., Usvyat D., Schuetz M., Dedkov Y. & Paulus B. On the physisorption of water on graphene: a CCSD(T) study. Phys. Chem. Chem. Phys. 13, 12041–12047 (2011). 21625710 10.1039/c1cp20609e

[b14] Ambrosetti A. & Silvestrelli P. L. Adsorption of Rare-Gas Atoms and Water on Graphite and Graphene by van der Waals-Corrected Density Functional Theory. J. Phys. Chem. C 115, 3695–3702 (2011).

[b15] Kim D., Pugno N. M., Buehler M. J. & Ryu S. Solving the Controversy on the Wetting Transparency of Graphene. Sci. Rep. 5, 15526 (2015). 26496835 10.1038/srep15526 PMC4620452

[b16] Driskill J., Vanzo D., Bratko D. & Luzar A. Wetting transparency of graphene in water. J. Chem. Phys. 141, 18C517 (2014). 10.1063/1.4895541 25399182

[b17] Checco A., Rahman A. & Black C. T. Robust Superhydrophobicity in Large-Area Nanostructured Surfaces Defined by Block-Copolymer Self Assembly. Adv. Mat. 26, 886–891 (2014). 10.1002/adma.201304006 24142578

[b18] Li X. *et al.* Large-Area Synthesis of High-Quality and Uniform Graphene Films on Copper Foils. Science 324, 1312–1314 (2009). 19423775 10.1126/science.1171245

[b19] Asadi K. *et al.* Up-Scaling Graphene Electronics by Reproducible Metal-Graphene Contacts. ACS Appl. Mater. Interfaces 7, 9429–9435 (2015). 25901791 10.1021/acsami.5b01869

[b20] Kim K. S. *et al.* Large-scale pattern growth of graphene films for stretchable transparent electrodes. Nature 457, 706–710 (2009). 19145232 10.1038/nature07719

[b21] Kobayashi T. *et al.* Production of a 100-m-long high-quality graphene transparent conductive film by roll-to-roll chemical vapor deposition and transfer process. Appl. Phys. Lett. 102, 023112 (2013).

[b22] Lin Y.-C. *et al.* Graphene Annealing: How Clean Can It Be? Nano Lett. 12, 414–419 (2012). 22149394 10.1021/nl203733r

[b23] Wood J. D. *et al.* Annealing free, clean graphene transfer using alternative polymer scaffolds. Nanotechnology 26, 055302 (2015). 25580991 10.1088/0957-4484/26/5/055302

[b24] Reserbat-Plantey A. *et al.* Strain Superlattices and Macroscale Suspension of Graphene Induced by Corrugated Substrates. Nano Lett. 14, 5044–5051 (2014). 25119792 10.1021/nl5016552

[b25] Ishigami M., Chen J. H., Cullen W. G., Fuhrer M. S. & Williams E. D. Atomic structure of graphene on SiO_2_. Nano Lett. 7, 1643–1648 (2007). 17497819 10.1021/nl070613a

[b26] Salehi-Khojin A. *et al.* Polycrystalline Graphene Ribbons as Chemiresistors. Adv. Mat. 24, 53–57 (2012). 10.1002/adma.201102663 22113971

[b27] Quéré D. Wetting and roughness. Annu. Rev. Mater. Res. 38, 71–99 (2008).

[b28] Cassie A. B. D. & Baxter S. Wettability of porous surfaces. T. Faraday. Soc. 40, 0546–0550 (1944).

[b29] Py C. *et al.* Capillary Origami: Spontaneous wrapping of a droplet with an elastic sheet. Phys. Rev. Lett. 98, 156103 (2007). 17501365 10.1103/PhysRevLett.98.156103

[b30] Patra N., Wang B. & Kral P. Nanodroplet Activated and Guided Folding of Graphene Nanostructures. Nano Lett. 9, 3766–3771 (2009). 19852466 10.1021/nl9019616

[b31] Israelachvili J. Intermolecular and Surface Forces. (Academic Press, 1992).

[b32] Shih C.-J., Strano M. S. & Blankschtein D. Wetting translucency of graphene. Nature Mat. 12, 866–869 (2013). 10.1038/nmat3760 24056845

[b33] Leroy F., Liu S. & Zhang J. Parametrizing Nonbonded Interactions from Wetting Experiments via the Work of Adhesion: Example of Water on Graphene Surfaces. J. Phys. Chem. C 119, 28470–28481 (2015).

[b34] Sforzini J. *et al.* Approaching Truly Freestanding Graphene: The Structure of Hydrogen-Intercalated Graphene on 6H-SiC(0001). Phys. Rev. Lett. 114, 106804 (2015). 25815955 10.1103/PhysRevLett.114.106804

[b35] Britnell L. *et al.* Field-Effect Tunneling Transistor Based on Vertical Graphene Heterostructures. Science 335, 947–950 (2012). 22300848 10.1126/science.1218461

[b36] Chen S. *et al.* Oxidation Resistance of Graphene-Coated Cu and Cu/Ni Alloy. ACS Nano 5, 1321–1327 (2011). 21275384 10.1021/nn103028d

[b37] Berman D., Erdemir A. & Sumant A. V. Few layer graphene to reduce wear and friction on sliding steel surfaces. Carbon 54, 454–459 (2013).

[b38] Coraux J. *et al.* Air-Protected Epitaxial Graphene/Ferromagnet Hybrids Prepared by Chemical Vapor Deposition and Intercalation. J. Phys. Chem. Lett. 3, 2059–2063 (2012).

[b39] O’Hern S. C. *et al.* Selective Ionic Transport through Tunable Subnanometer Pores in Single-Layer Graphene Membranes. Nano Lett. 14, 1234–1241 (2014). 24490698 10.1021/nl404118f

