# Supplementary material for: Wettability of partially suspended graphene
Source: Sci Rep. 2016 Apr 13;6:24237. doi: 10.1038/srep24237 (PMC4829856; doi:10.1038/srep24237)
Supplement: Supplementary Information [file srep24237-s1.pdf]

Supplementary information

Wettability of partially suspended graphene

Thierry Ondarçuhu, [1*] Vincent Thomas, [1] Marc Nuñez, [1] Erik Dujardin, [1] Atikur Rahman, [2] Charles T. Black,[2] and Antonio Checco, [3*]

[1] Nanosciences group, CEMES-CNRS, 29 rue Jeanne Marvig, 31055 TOULOUSE (France)

[2] Center for Functional Nanomaterials, Brookhaven National Laboratory, Upton, NY, 11973 (USA)

[3] Condensed Matter Physics and Materials Science Department, Brookhaven National Laboratory, Upton, NY 11973 (USA)

Supplementary Information

SI1: Graphene wrinkles formation .... p. 2

SI2: Characterization of suspended graphene layers .... p. 3

SI3: Annealing procedure .... p. 5

SI4: Protocol for contact angle measurements on supported graphene layers .... p. 7

SI5: Influence of defects .... p. 10

SI6: Wettability of graphene multilayer and graphite....................................... p.12

References .... p. 13

SI1: Graphene wrinkles formation

The transfer method used in this study leads to the formation of wrinkled graphene layers. In order to get insight in the formation of the folds, we observed graphene layers during all steps of the process. The pattern of folds is not correlated to the grain boundaries of the copper foil supporting the graphene layer at the initial stage. Moreover, PMMA-assisted transfer of the same graphene sample did not show the presence of any wrinkle, indicating that the folding occurs latter in the process. Indeed, it was observed that the graphene layer is already folded when lying at the liquid interface (see Fig SI1) showing that folds do not result from the drying of the liquid layer on the substrate. The wrinkling therefore occurs at the liquid interface, due to surface tension effects when the copper foil is etched away. Indeed, the first millimeter in the periphery of the graphene layer is fully crumpled which is useful to visualize the graphene sheet floating at the liquid interface.

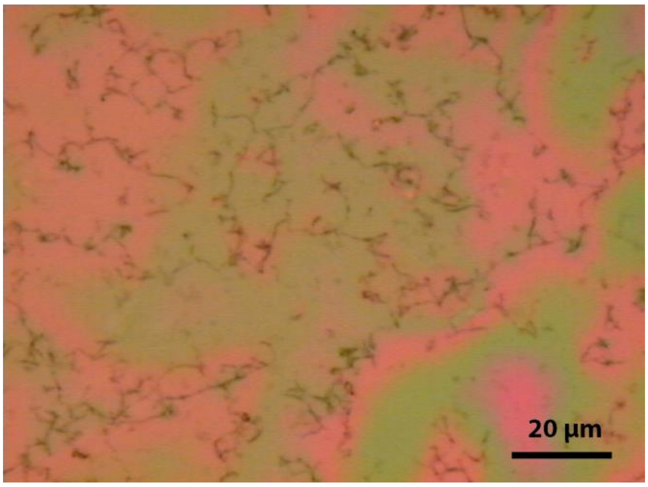

Fig SI1: Optical micrograph of a graphene layer floating on a liquid film above a hydrophilic flat SiO_2 substrate. Colors are due to interference fringes in the thin liquid film. Black features are folds of the graphene layer.

SI2: Characterization of suspended graphene layers

The partially supported graphene layers were characterized by SEM and AFM. The patterns of folds present the same characteristics as the ones observed on flat substrates. They are clearly evidenced in the images (see Fig. SI2a, SI2c and SI2d), together with the holes in the layer. The main difference with flat substrates lies in the occasional presence, on patterned substrates, of short cracks that propagate following the paths between posts (see Fig. SI2b). For substrates with conical texture ($\phi < 15\%$), the density of defects may increase significantly and reach 8% for the sharper surface used ($\phi = 6\ \%$) as shown in Fig. SI2e.

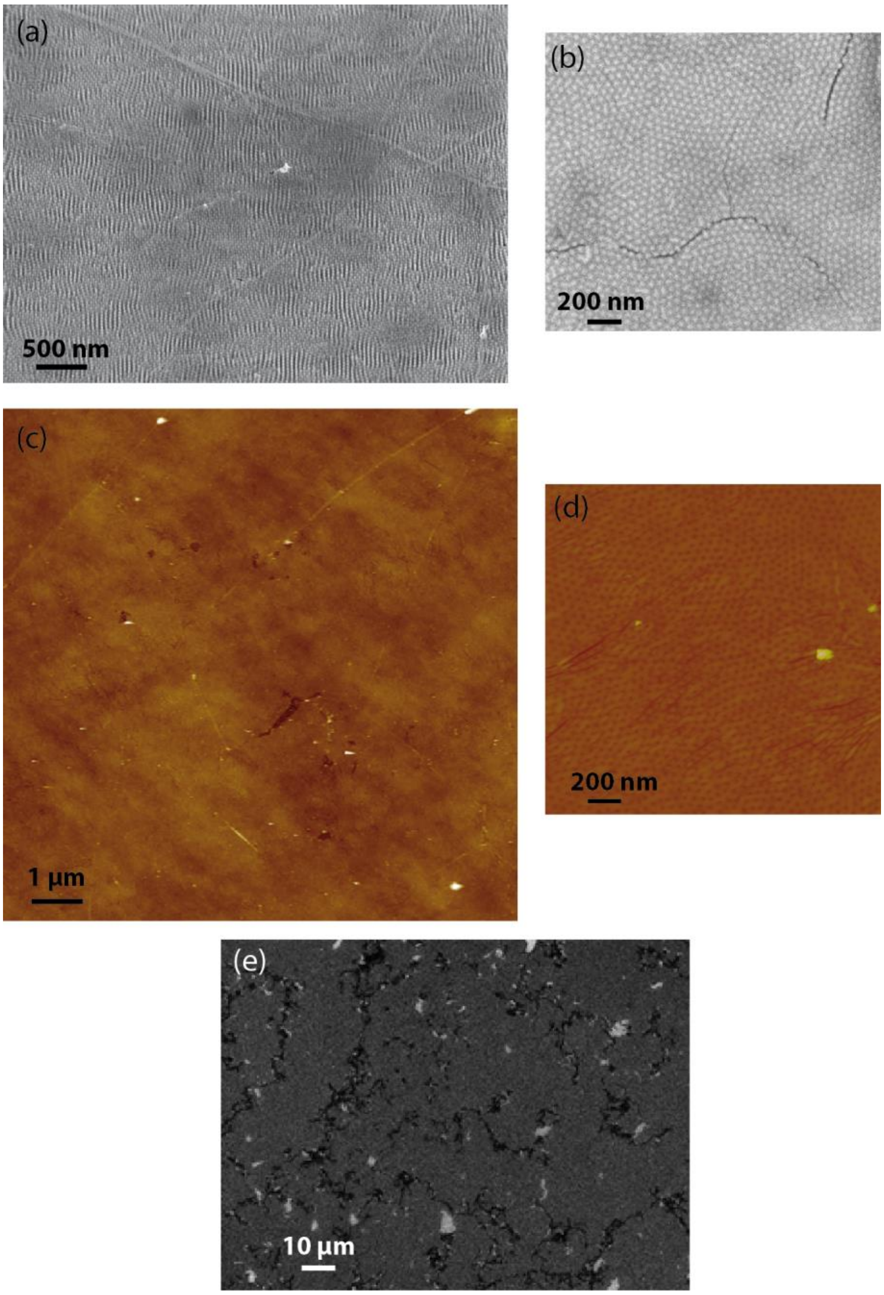

Fig SI2: (a) SEM image of a graphene monolayer deposited on a substrate with nailhead posts (for better contrast the sample was tilted by54°). (b) SEM image on an area where the layer presents some cracks following the pillars pattern. (c) Large scale AFM image of a partially suspended monolayer where holes and folds are clearly visible. (d) Zoom on a flat area. (e) SEM image of a graphene layer deposited on a substrate with conical texture ($\phi = 6\%$). The contrast was enhanced to visualize the holes in the layer which appear bright in the image and represents 8% of the total area.

SI3: Annealing procedure

Prior to any contact angle measurements, the samples were cleaned by annealing under a Ar/H_2 atmosphere. The samples were heated up to 350 °C following a ramp of 5°C/min, under a argon flux of 300 sccm, and kept at this temperature during 4 hours with an additional flux of hydrogen (75 sccm). The oven was naturally cooled down to ambient temperature under Ar flux. Even though the optical imaging reveals the removal of some black particles already present on the copper foil, the effect of the annealing process on the graphene quality was further assessed by high resolution TEM (HR-TEM) on graphene layers deposited using the resist-free procedure on TEM grids. The images before and after annealing are reported on Fig. SI3a-b together with diffraction patterns. No structure is visible before annealing whereas the crystalline structure of the graphene clearly shows up in the TEM micrographs and diffraction after treatment, demonstrating the efficiency of the Ar/H_2 annealing.

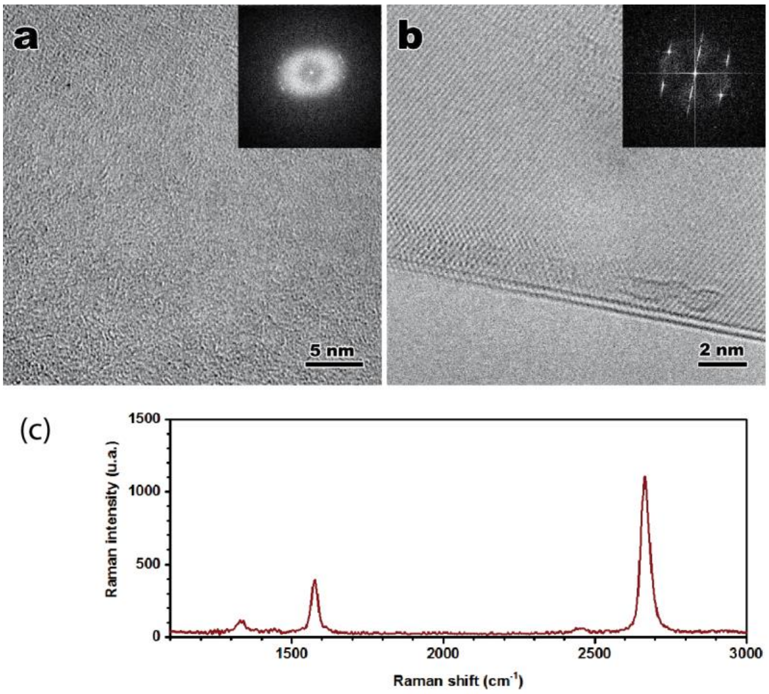

Fig SI3: HRTEM image and diffraction pattern (inset) of a graphene monolayer: (a) before annealing; (b) after annealing; (c) Raman spectrum of a supported graphene layer.

Micro-Raman spectra were acquired on a Horiba Xplora-MV2000 spectrometer in a 1-µm focal spot area exempt of visible wrinkle (Figure SI3c). Two intense peaks are recorded at 1580 cm^{-1} and 2670 cm^{-1} that correspond to the G and 2D bands of graphene respectively. Two weaker peaks at 1330 cm^{-1} and 2450 cm^{-1} are the first order D band and the second order D+D'' band,[1,2] which characterize defects in the layer. The areal ratio of the 2D and G bands ($\frac{A_{2D}}{A_G}\sim 4-5$) together with the single Lorentzian shape of the 2D band are strong indications of a single monolayer. As expected, micro-Raman spectra measured on wrinkles exhibit a much more intense D band due to a higher density of defects in these regions.

SI4: Protocol for contact angle measurements on supported graphene layers

The contact angle measurements were performed on a Kruss DSA100 goniometer. In order to optimize the protocol to achieve contact angle measurements of water on graphene layers, we transferred on SiO_2/Si flat samples, graphene layers from three different commercially available graphene sources (two from Graphene Supermarket Inc. USA and one from Graphenea, SP) grown on copper surfaces by chemical vapor deposition (CVD). After transfer the samples were cleaned using the annealing procedure described in the previous section.

Advancing and receding contact angles were measured on several graphene regions, immediately after the reductive annealing and for a few hours afterwards (see Figure SI4) in order to probe the dynamics of airborne contaminants re-adsorption. The results show no influence of the graphene source. Importantly, the measurements on flat substrates reveal an increase of the contact angle with time, in agreement with recent reports.[3,4] In particular, the advancing contact angle increases from 68° ± 1 ° reaching a plateau at 85° ± 2° when exposed to ambient air, in agreement with the results of Li *et al.*.[3] However, we have found that the wettability change occurs on a time scale of ca. 5 hours rather than 1h, as reported in ref. 2a, provided that the cleaned samples are kept under nitrogen atmosphere at all time. In agreement with recent AFM force measurements,[5] this evolution of the advancing angle is attributed to the decrease in effective surface energy as water and airborne hydrocarbon contaminants adsorb on graphene. For these reasons, all the contact measurements reported here were performed within ten minutes from the end of annealing process. Conversely, the receding contact angle was found not to vary significantly (θ_{rec}= 45° ± 2°) after the annealing process, suggesting that this quantity is dominated by pinning of the receding contact line on anchoring defects,[6] which are present after the transfer of graphene but do not evolve in time.

Interestingly, the receding contact angle found here is identical to the one reported in ref. 2a on wrinkle-free graphene obtained by PMMA-assisted transfer, thus indicating that the wrinkles generated by the resist-free transfer method do not modify significantly the anchoring of water droplets on graphene.

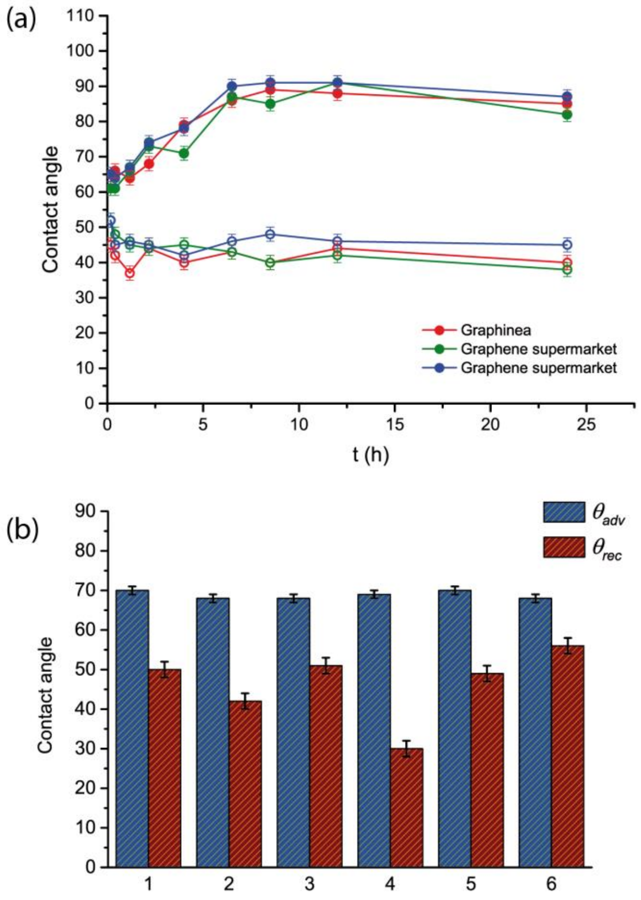

Fig SI4: (a) Advancing (filled circles) and receding (empty circles) water contact angles of supported graphene monolayers from three different sources, as a function of the time after the annealing process. (b) Advancing (blue bars) and receding (red bars) water contact angles of 6 different supported Graphene Supermarket graphene monolayers.

The repeatability of the contact angle measurements was checked by performing identical experiments on six different layers transferred on flat Si/SiO_2 surfaces pre-cleaned by sulfuric

acid and hydrogen peroxide solution. The results show that the value of the advancing contact angle is highly reproducible (standard deviation < 1°) while receding contact angle values are more spread (s.d. > 9°). This is also consistent with the conclusions of Raj *et al.*[6] and suggests that the advancing contact angle provides a more reliable measure of the intrinsic wettability of graphene compared to the receding angle, which is more influenced by monolayer defects. For this reason, we only report advancing contact angles measurements.

SI5: Influence of defects

In order to assess the influence of defects in the graphene layer, we considered a graphene layer with a contact angle θ_G assumed independent of the underlying substrate and with a density of defects (holes) Φ_d. The contact angle of the defective layer can then be calculated using a Cassie-Baxter equation using the substrate contact angle θ_S for the defects. It reads

$$\cos\theta = (1 - \Phi_d)\cos\theta_G + \Phi_d \cos\theta_S \quad (1)$$

On Fig. SI5, the expression given by Eq. (1) is plotted for two different density of defects namely Φ_d=2% and 8%. As expected the influence of defects increases with defects density. For $\Phi_d = 2\%$ which corresponds to the majority of samples studied, the variation of contact angle is negligible. On sharp textures, the density of defects increases up to $\Phi_d = 8\%$ which may explain part of the evolution of contact angle measured experimentally.

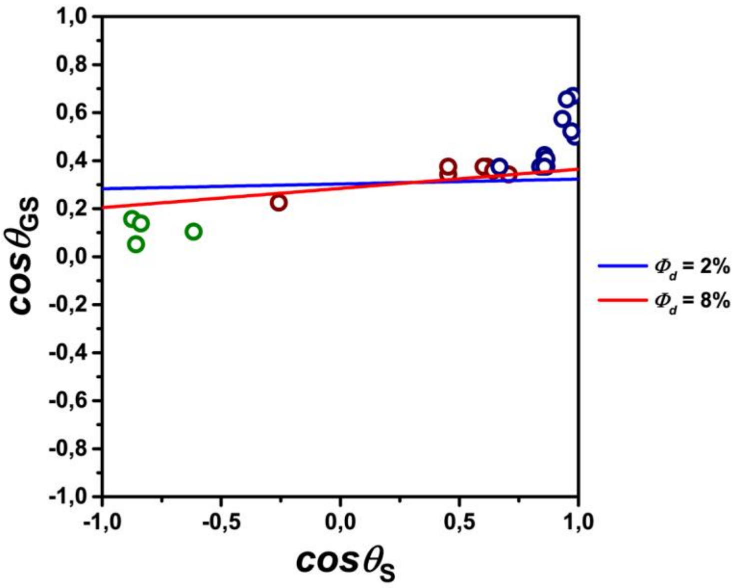

Figure SI5. Plot of the experimental data (dots) and of the predicted curves for three different densities of defects (Eq. (1)).

The variation of contact angle $\Delta\theta_d$ between graphene suspended on water ($\cos\theta_S = 1$) and air ($\cos\theta_S = -1$) associated to the only presence of defects can be estimated using Eq. (1). It reads:

$$\Delta\theta_d = -\frac{2\phi_d}{\sin\theta} \qquad (2)$$

It gives $\Delta\theta_d \approx -3°$ for $\Phi_d = 2$ % and $\Delta\theta_d \approx -10°$ for $\Phi_d = 8$ % which can explain only a fraction of the $\Delta\theta_{GS} \approx -24°$ value measured experimentally.

SI6: Wettability of multilayer graphene and graphite

In a rough approximation, the wettability of multilayer graphene can be estimated graphically Indeed, the contact angle on n+1 layers can be determined from the contact angle on n layers using the $cos\theta_{GS} = f(cos\theta_S)$ relation deduced experimentally. This leads to $cos\theta_{(n+1)GS} = f(cos\theta_{nGS})$. $cos\theta_{(n+1)GS}$ can then be used to compute $cos\theta_{(n+2)GS}$. This can be simply obtained graphically using the diagonal of the graph as shown on Figure SI6a starting from a suspended single monolayer. The same could be obtained for multilayer graphene floating on water, starting from $cos\theta_S = 1$, or from any situation. In Figure SI6b are reported the contact angles as a function of the number of layers in both situations. It shows that both values converges rapidly towards a value which corresponds to the contact angle on HOPG i.e the stack of an infinite number of graphene layers. This convergence is rather fast since the contact angle value for a 3-layers (4-layers) configuration approaches the one of HOPG value within 0,7° (0,14°), respectively.

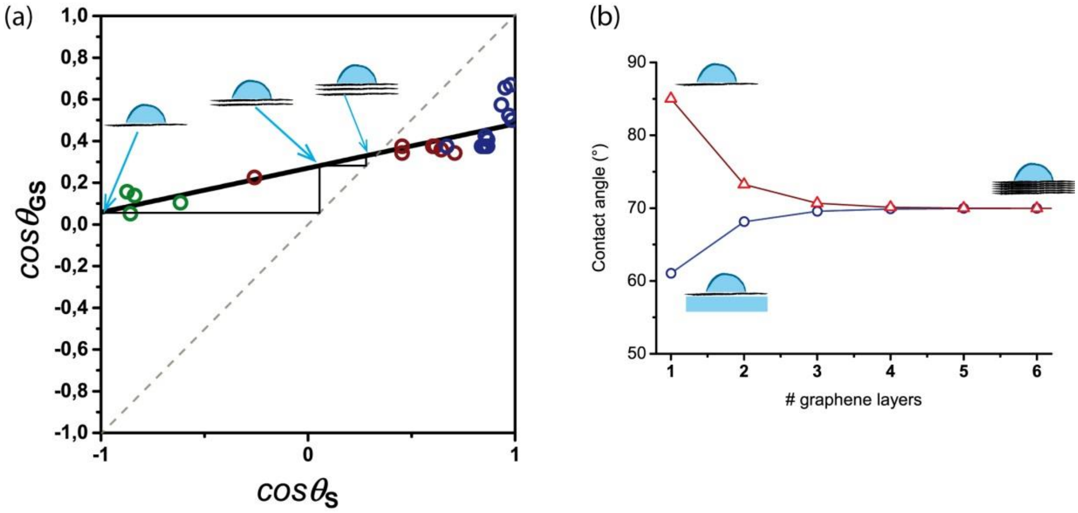

Figure SI6. (a) Graphical construction of the contact angle on multi-layers graphene starting form a single suspended graphene layer. (b) Contact angle of water as a function of the number of graphene layers suspended in red triangles and floating on water in blue circles.

References

1. May, P. *et al.* Signature of the two-dimensional phonon dispersion in graphene probed by double-resonant Raman scattering. *Phys. Rev. B* **87**, 075402, (2013).
2. Narula, R. & Reich, S. Probing LO phonons of graphene under tension via the 2D ' Raman mode. *Phys. Rev. B* **87**, 115424, (2013).
3. Li, Z. *et al.* Effect of airborne contaminants on the wettability of supported graphene and graphite. *Nature Mat.* **12**, 925-931, (2013).
4. Kozbial, A. *et al.* Understanding the intrinsic water wettability of graphite. *Carbon* **74**, 218-225, (2014).
5. Lai, C.-Y. *et al.* A nanoscopic approach to studying evolution in graphene wettability. *Carbon* **80**, 784-792, (2014).
6. Raj, R., Maroo, S. C. & Wang, E. N. Wettability of Graphene. *Nano Lett.* **13**, 1509-1515, (2013).
